# Research on the Physics–Intelligence Hybrid Theory Based Dynamic Scenario Library Generation for Automated Vehicles

**DOI:** 10.3390/s22218391

**Published:** 2022-11-01

**Authors:** Yufei Zhang, Bohua Sun, Yaxin Li, Shuai Zhao, Xianglei Zhu, Wenxiao Ma, Fangwu Ma, Liang Wu

**Affiliations:** 1 State Key Laboratory of Automotive Simulation and Control, Jilin University, Changchun 130025, China; 2 China Automotive Technology & Research Center (CATARC) Co., Ltd., Tianjin 300399, China; 3 College of Intelligence and Computing, Tianjin University, Tianjin 300072, China

**Keywords:** automated vehicles, dynamic scenario generation, scenario boundary evaluation, physics–intelligence hybrid theory, system identification, reinforcement learning

## Abstract

The testing and evaluation system has been the key technology and security with its necessity in the development and deployment of maturing automated vehicles. In this research, the physics–intelligence hybrid theory-based dynamic scenario library generation method is proposed to improve system performance, in particular, the testing efficiency and accuracy for automated vehicles. A general framework of the dynamic scenario library generation is established. Then, the parameterized scenario based on the dimension optimization method is specified to obtain the effective scenario element set. Long-tail functions for performance testing of specific ODD are constructed as optimization boundaries and critical scenario searching methods are proposed based on the node optimization and sample expansion methods for the low-dimensional scenario library generation and the reinforcement learning for the high-dimensional one, respectively. The scenario library generation method is evaluated with the naturalistic driving data (NDD) of the intelligent electric vehicle in the field test. Results show better efficient and accuracy performances compared with the ideal testing library and the NDD, respectively, in both low- and high-dimensional scenarios.

## 1. Introduction

With the continuous improvement of intelligence levels, the driving manipulation body of automated vehicles is transforming from human divers to an automated system [1]. Therefore, the testing and evaluation system (TES) of automated vehicles must be developed to verify their performance comprehensively and reliably [2]. A clear and definitive conclusion has been inferred in many previous studies that only billions of miles or scenarios for automated vehicles can achieve a better verification performance than those for human drivers at a 95% confidence level [3,4]. Currently, the implementation of the TES mainly consists of three methods, the offline simulation test, the X-in-the-loop test, and the field test [5]. Given the requirements of the TES, such as test consistency, reliability, safety, etc., and the constraints, such as the test cost, period, efficiency, etc., it is too hard to conduct the TES in the field test [6]. In contrast, the simulation-based offline and X-in-the-loop tests are becoming the inevitable trend for the TES with their advantages on test efficiency, cost, consistency, etc. [7,8]. For a specific operational design domain (ODD), the simulation-based TES conducts billions of miles or scenarios to verify the system performance of automated vehicles [9]. However, large-scale scenarios are still a serious challenge for the testing efficiency of the simulation-based testing system [10]. What is more, typical naturalistic driving data (NDD) brings reliable but low-coverage test results, while the ideal scenario space data (ISSD) brings high-coverage but low-confidence test results [11,12]. Therefore, the generation method of the dynamic scenario library (DSL) in the simulation-based system is the core technology in the TES, which consists of three components with limitations at present, the parameterized scenario specification, the evaluation system of the DSL, and the scenario searching method.

As the description basis and generation foundation of the DSL, the scenario element definition-based parameterized scenario specification (PSS) aims to describe real scenarios and construct virtual scenarios based on various parametric models [13]. Therefore, the scenario deconstruction logic (SDL) and its corresponding parametric models show significant roles in the PSS. The extraction method of scenario elements and the analysis of their spatio-temporal coupling mechanism constitute key techniques in the SDL, and the multiple heterogeneous systems with various scenario elements and their coupling laws achieve parametric scenario models [14,15,16]. Layering scenarios and extracting their elements are two typical ways in the SDL [17,18]. The ISO and ASAM deconstruct scenarios into several levels with the representation of OpenX [19,20]. The element level specifies the types and forms of the scenario elements in detail, the structure level is comprised of the element classification and scenario models, and the semantic level interprets the perceptive mode and diving capability between the ego vehicle and background vehicles [21]. In contrast, data learning and logic classification methods, such as classification trees, data clustering, or expert experience database, are developed in scenario element extraction [22,23]. Datasets consisting of scenario elements are generally designed for the specific ODD, thus combinations of the element division, classification, and perception are integrated into scenario datasets. Methods in the SDL show consistent results that both internal elements and their associations and constraints should be considered. However, an elaborate element divide still needs to be developed in the scenario-level system and element dimensions need to be optimized as a set of independent elements [24,25]. Therefore, the dimension optimization of scenario elements is the key to avoiding explosive and redundant scenario space and needs to be developed in depth.

The long-tail effect dilemma in the testing and evaluation system for automated vehicles brings a huge amount of the testing mileages for each system vulnerability, and the scenario boundary evaluation system (SBES) for the acceleration test gets wide attention [26,27]. The SBES can be regarded as an optimization problem in the specific ODD or performance index, and boundary scenarios are obtained by approximating a given optimization function with efficient solvers or algorithms [28]. The optimization target function-based boundary searching method, such as the collision cone index, the emergency evaluation model, or the criticality index, seeks the scenario boundaries by searching index spaces [29,30]. The global optimal solution algorithm-based boundary solution aims to converge the target function to the global optimal solution rapidly and accurately with machine learning or optimization algorithms, such as particle swarm optimization, random tree search algorithm, or genetic algorithm [31,32]. In order to improve the tacit understanding degree between test scenarios and corresponding test objects, online adaptive boundary generation methods are developed, such as reinforcement learning with the Monte Carlo tree search [33]. In addition, the scenario derivation algorithm-based boundary extension method is developed as well based on a neural network framework, and boundaries are identified and predicted in the online platform [34]. What is more, the NDD-based SBES is the key technique to construct boundary datasets as well. Datasets for SBES, such as KITTI, Cityscape, and ApolloScape are developed and data-driven algorithms, such as the Gaussian process classification or long short-term memory, are used to optimize the datasets [35,36]. However, the boundary optimization method and the NDD construction system are developed independently, and a significant number of low-probability scenarios are included in the scenario boundary datasets due to the lack of verification from NDD [37,38]. Therefore, both the scenario occurrence probability in the real world and its physical critical degree should be considered in the boundary evaluation system to guarantee the rationality of the boundary function, and their combination function should be designed in detail.

Scenario-searching methods aim to extract effective scenarios from original scenario datasets and constitute the generation system of the DSL together with the SBES [39]. In view of the system properties of the DSL, such as time-varying, nonlinear, multi-source, and heterogeneous, a variety of basic theories have been tried to implement scenario search methods [40]. NDD constructed from multi-source sensors, such as the Kunlun project of China, KITTI, and the BDD100K from the University of California Berkeley is generally regarded as the most primitive and direct generation method for DSL [41]. However, testing periods and costs are greatly increased due to the redundant and invalid data, and limitations appear in the test effectiveness given the coverage of the specific NDD [42,43]. To solve these issues, artificial intelligence or physical model-based scenario elimination methods are developed, such as the worst-case scenario evaluation, the importance sampling accelerated evaluation, and the spring-mass model-based crash scenario evaluation [44,45,46]. Low-dimensional scenario generation methods are established but not suitable for the high-dimensional and the generation process is usually intractable. What is more, some data traversal-based elimination methods have a weak role in the testing period and cost due to their traversal mode in the scenario generation. To avoid these problems, several preliminary attempts are conducted for the adaptive generation of high-dimensional scenarios [47]. The specialized adaptive search algorithm-based scenario generation method is designed for the extraction of emergency scenarios but has limitations in the elimination of redundant scenarios [48]. The deep learning-based scenario reconstruction method establishes and tracks dynamic scenarios accurately with visual data sequences and its limitations exist in the over-reliance on visual data and weak compatibility with other sources [49]. The PEGASUS project extracts the risky scenarios according to the definition of all feasible scenarios risk from ISO 26262 but lacks data from the real world [50]. It can be seen that the generation methods of high-dimensional scenarios for the DSL are still immature and their relationships with the low one need to be analyzed. As a result, the scenario searching method suitable for both low- and high-dimension scenarios should be developed in-depth and a general generation framework of the dynamic scenario library should be established.

Motivated by the above-mentioned observations, the physics–intelligence hybrid theory (PIHT) based dynamic scenario library generation method for automated vehicles is proposed. The main contributions of this research are as follows:

(1) A general generation framework of the dynamic scenario library is established based on the physics–intelligence hybrid theory to improve the testing efficiency and accuracy for automated vehicles. Three components, scenario parameterization, long-tail functions, and critical scenario generation, are specified and adopted to generate both low- and high-dimensional scenarios.
(2) The dimension optimization method-based parameterized scenario specification is designed. Scenarios are extracted as a set of multiple heterogeneous elements and the analytic hierarchy process is used in the dimension optimization.
(3) Long-tail function construction methods are proposed to obtain clear and reasonable scenario boundaries for both low- and high-dimensional scenarios. The NDD-based ISSD is classified and clustered, and the labeled scenario space boundary is mapped with the critical degree.
(4) Critical scenario searching methods are proposed to generate critical scenarios in the dynamic scenario space. The combination method of node optimization and sample expansion is proposed for the generation of low-dimension scenarios and the reinforcement learning method for that of the high-dimension scenarios.

## 2. Framework of the Dynamic Scenario Generation Method for Automated Vehicles

The scenario system for the TES of automated vehicles can be regarded as a dynamic description of automated vehicles and their surrounding dynamic and static environment over a period of time. In particular, it is an organic combination of the automated vehicle and its surrounding environment. Given scenario dynamic properties of time-varying, nonlinear, multi-source, and heterogeneous, scenarios in the TES are infinite and diverse. In order to obtain quantitative and accurate testing and evaluation results, the TES is generally developed for a specific ODD, which can be defined as a whole testing maneuver that the automated vehicle is developed to function [50]. Taking that purpose as a starting point, generation methods for dynamic low-dimensional scenarios (LD-Scenarios) consisting of discrete scenario elements and high-dimensional scenarios (HD-Scenarios) combining the continuous observation sequences and discrete scenario elements are major issues in the research of TES. Generating a statistically unbiased scene DSL without traversal is the key point for the acceleration testing method in the LD-Scenario research, and the accurate and effective searching and optimal set-solving methods are that for the HD-Scenarios. What is more, the consistency and accuracy of testing results and the relevance of the logic between LD-Scenarios and HD-Scenarios are significant for the testing and evaluation of the specific ODD as well.

**Figure 1:**
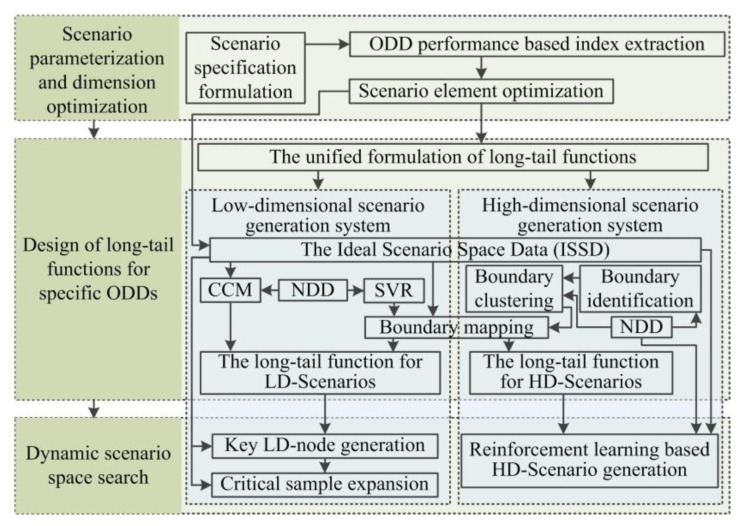
Framework of the dynamic scenario generation system.

In order to generate an accurate and effective DSL in the TES of automated vehicles, the general framework of the dynamic scenario generation system is proposed and shown in Figure 1. It consists of three components, the module for the scenario parameterization and dimension optimization, the design module of long-tail functions for specific ODDs, and the module for the dynamic scenario space search. The dimension optimization method based on parameterized scenario specification is proposed to obtain the general scenario formulation and optimized scenario elements. Long-tail functions are designed as optimization boundaries considering the rationality of modeling, the uniformity of sampling, and the probability of occurrence in the real world. The dynamic scenario space search is proposed to adapt to both LD and HD-Scenarios. The PIHT, which can be defined as the joint combination theory of the physical mechanism model and data learning algorithm, is proposed in the generation method of the DSL given the physical attributes of the DSL and their multi-source heterogeneous data schema. The physical mechanism and data modal are analyzed in detail and methods of physical models and machine learning are combined compactly and reasonably in the PIHT. Taking the above-mentioned into consideration, the general framework is designed, and technical details are developed in the following parts.

## 3. Physics–Intelligence Hybrid Theory Based Dynamic Scenario Generation System

### 3.1. The Dimension Optimization Method-Based Parameterized Scenario Specification

In the parameterized scenario specification module, scenarios are decoupled and formulated into typical dynamic elements and static elements first in order to achieve a clear and reasonable scenario specification. Then, the system performance in the TES and their indexes are analyzed and formulated for both LD and HD-Scenarios based on basic test tasks of ODDs. Finally, in order to avoid explosive and redundant scenario space, scenario elements are optimized with the analytic hierarchy process (AHP) and the optimized scenario formulation with the quantized performance specification is established.

#### 3.1.1. The Scenario Specification Formulation

**Figure 2:**
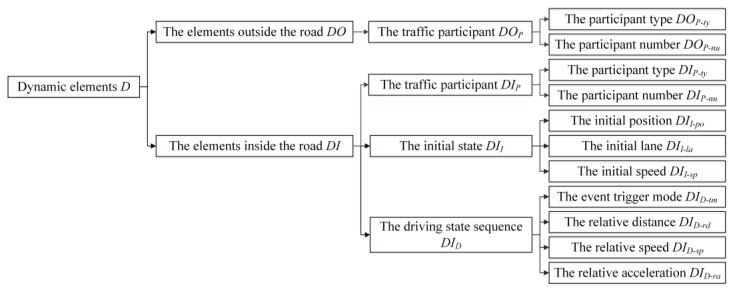
The classification of dynamic elements.

Scenario elements are basic units in the TES and can be divided into dynamic elements *D* and static elements *S*. On the basis of the geographical location of scenario elements, both dynamic elements and static elements can be further divided into the elements located outside and inside the road. Dynamic elements outside the road *DO* mainly include the traffic participant *DO_P_*, which consists of the participant type *DO_P-ty_* (such as the bicycle, the pedestrian, and other creatures) and the participant number *DO_P-nu_*. Dynamic elements inside the road *DI* mainly include the traffic participant *DI_P_*, the initial state *DI_I_*, and the driving state sequence *DI_D_* of the traffic participant. *DI_P_* contains the participant type *DI_P-ty_* (such as the passenger car, the truck, the motor tricycle, and other vehicles) and the participant number *DI_P-nu_*. *DI_I_* contains the initial position *DI_I-po_*, the initial lane *DI_I-la_*, and the initial speed *DI_I-sp_*. *DI_D_* contains the event trigger mode *DI_D-tm_* (such as the distance mode or the time mode), the relative distance *DI_D-rd_*, the relative speed *DI_D-rs_* and the relative acceleration *DI_D-ra_*. The classification of dynamic elements is shown in Figure 2.

**Figure 3:**
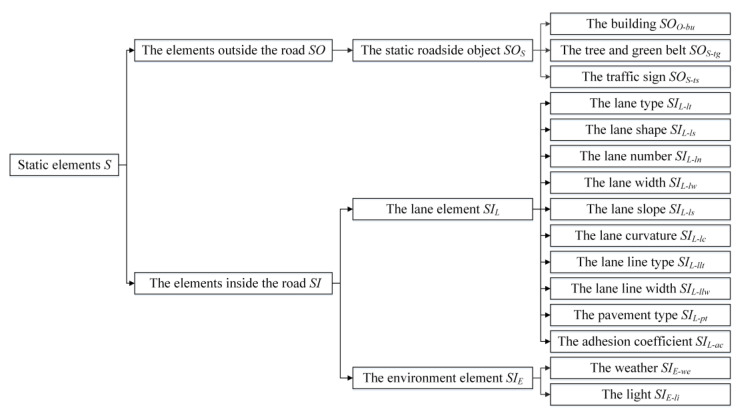
The classification of static elements.

Static elements outside the road *SO* mainly include the static roadside object *SO_S_*. *SO_S_* contains the building *SO_S-bu_*, the tree and green belt *SO_S-tg_*, and the traffic sign *SO_S-ts_*. The static elements inside the road *SI* mainly include the lane element *SI_L_* and the environment element *SI_E_*. *SI_L_* contains the lane type *SI_L-lt_* (such as the expressway, the urban road, and the rural road), the lane shape *SI_L-ls_* (such as the straight road, the curve, and the intersection), the lane number *SI_L-ln_*, the lane width *SI_L-lw_*, the lane slope *SI_L-ls_*, the lane curvature *SI_L-lc_*, the lane line type *SI_L-llt_*, the lane line width *SI_L-llw_*, the pavement type *SI_L-pt_* (such as the asphalt pavement and the cement concrete pavement) and the road adhesion coefficient *SI_L-ac_*. *SI_E_* contains the weather *SI_E-we_* (such as sunny, cloudy, rainy, snowy, foggy, and windy), and light *SI_E-li_* (such as sufficient, dim, and changeable). The classification of static elements is shown in Figure 3.

#### 3.1.2. The Performance Index Extraction Method

The performance metrics of the automated vehicle include safety, functionality, mobility, and rider comfort. Safety is the biggest one, which can be measured by the index of the accident rate. Functionality represents whether the automated vehicle can complete a specific task successfully and can be measured by the index of the failure rate. Mobility is used to reflect the flexibility of the automated vehicle when it is faced with a complex road structure and can be measured by the index of travel efficiency. Rider’s comfort measures the physical and psychological feelings of passengers and the quantitative index can be the jerk, or the root mean square value of the acceleration.

To estimate these indices of performance metrics, the event of interest is denoted as *E*, such as the accident in the safety evaluation and the failure in the functionality evaluation. To evaluate the mobility and rider’s comfort, the event of interest is proposed and can be defined as the event where the index is failed to reach a certain threshold. The occurrence probability of *E*, i.e., the performance index, is denoted as *p*(*E*), such as the accident rate. If the tested automated vehicle experiences *n* target scenarios and has *m* events of interest *E*, the performance index can be estimated as follow.

(1) $$p(E)\approx \frac{m}{n}$$

The theoretical justification is provided as follows. Target scenarios can be regarded as the following distribution *p*(*s*), i.e., *s_i_*~*p*(*s*), *i* = 1, …, *n*, and the performance index can be estimated as

(2) $$p(E)=\sum p(E|s)\cdot p(s)\approx \frac{1}{n}\sum_{i=1}^{n}p(E|s_{i}),where\;s_{i}∼p(s)\approx \frac{m}{n}$$

According to Bernoulli’s law of large numbers, the required test number *n* should be large enough to obtain a reasonable estimation accuracy.

#### 3.1.3. The AHP-Based Scenario Element Optimization Method

The importance weight of dynamic elements and static elements can be determined by AHP which is an effective method to solve complex and fuzzy evaluation problems, especially for problems that are difficult to be analyzed quantitatively [51]. The logical structure of the classification of scenario elements has clarified the subordination relationships of dynamic elements and static elements, so it can be used as the hierarchical structure model in AHP. The importance of every two scenario elements at the same level can be compared by the scaling method to construct the judgment matrix. The content of the scaling method is shown in Table 1.

**Figure 4:**
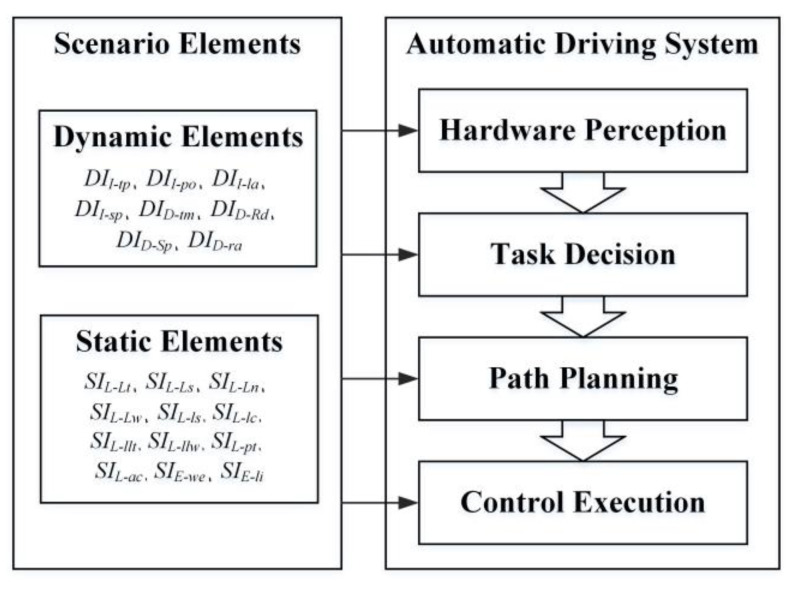
The content of ETM.

The effect transmission model (ETM) is constructed to measure the importance of scenario elements and obtain the relative ratio in the scaling method, as shown in Figure 4. The ETM quantifies the effect of each scenario element on the automated vehicles by calculating the passing times of the effect in all levels of the automated vehicles. When a scenario element has an effect on the previous level, the effect can be transferred to the current level automatically. At the same time, whether the scenario element has a direct effect on the current level also needs to be evaluated.

The effect transmission number is as follows.

(3) $$T(q)=\sum_{i=1}^{q}\left(N_{i}-1\right)$$

where *T*(*q*) is the transmission number of the effect, *q* denotes the number of attributes of the scenario element, for example, the scenario element *SI_E-we_* has six attributes such as sunny, cloudy, rainy, snowy, foggy, and windy. *N_i_* is the level number of automated vehicles affected by the attribute *i* of the scenario element. The corresponding relationship between the transmission number and the relative ratio is shown in Table 2.

The judgment matrix is constructed with the relative ratio. By solving the maximum eigenvalue and its corresponding eigenvector of the judgment matrix, the importance weights of scenario elements can be obtained by normalizing the eigenvector. In order to avoid the numerical singularity caused by the deviations in the judgment matrix, it is necessary to verify the matrix consistency. The testing indices are as follows.

(4) $$C_{I}=\frac{\lambda_{\max}-r}{r-1}$$

(5) $$C_{R}=\frac{C_{I}}{R_{I}}$$

where *C_I_*, *r, R_I_*, *C_R_* denote the consistency index, the order of the judgment matrix, the random consistency, and the consistency ratio, respectively. The judgment matrix meets the consistency when *C_R_* < 0.1. The value of *R_I_* is shown in Table 3.

### 3.2. Long-Tail Function Construction Methods

The design module of long-tail functions for specific ODDs integrates system characteristic of both LD and HD-Scenarios and the adaptive long-tail function set are established. The unified formulation of long-tail functions are designed based on the optimized scenario formulation and the physical features and probability of occurrence in the real world are taken into consideration. The ideal scenario space data (ISSD) is established as a uniform sample space and used for the function design and space search. In the LD-Scenario generation system, the convex combination method (CCM) is proposed to solve the occurrence probability with ISSD and NDD, and the support vector regression (SVR)-based boundary classification method is used to divide NDD into a typical degree of system performance. In the HD-Scenario generation system, the Hammerstein identification model-based boundary identification process is proposed as the physical model and the based boundary ant colony clustering method is used to cluster NDD into several datasets. Classification results of both LD and HD-Scenarios are mapped with physical conditions in the real world and the corresponding datasets are matched with clear physical boundaries. Finally, the occurrence probability and the typical degree of scenario performance are solved to construct long-tail functions.

#### 3.2.1. The Unified Formulation of the Long-Tail Function

Long-tail functions are constructed to obtain the scenario criticality from the ISSD for automated vehicles. Currently, most studies focus on selecting challenging scenarios from ISSD as the critical scenarios for performance evaluations. However, the occurrence probability of these selected scenarios is usually ignored. Some scenarios obtained by this method may not occur at all or have weak occurrence probabilities, which cannot reflect the real situation on the road. To make the selected scenarios have certain practical significance, a new principle with the combination of the challenge degree and occurrence probability is proposed. Inspired by the performance index estimation, the long-tail function is defined as follows.

(6) V(x)=C(x)⋅P(x)

where *V*(*x*) denotes the criticality of the scenario in ISSD, *P*(*x*) denotes the occurrence probability of the scenario in ISSD and it can be obtained by NDD, *C*(*x*) denotes the challenge degree of the scenario for the automated vehicle. The definition indicates that scenarios with higher occurrence probability in the real world and higher challenges should have higher priority for automated vehicle evaluation. The long-tail function is as follows in the safety evaluation.

(7) $$V_{s}(x)=R_{s}(x)\cdot P_{s}(x)$$

where *V_s_*(*x*) denotes the criticality of the scenario, *R_s_*(*x*) denotes the risk degree of the scenario, *P_s_*(*x*) denotes the occurrence probability of the scenario. The long-tail function is as follows in the functionality evaluation.

(8) $$V_{f}(x)=D_{f}(x)\cdot P_{f}(x)$$

where *V_f_*(*x*) denotes the criticality of the scenario, *D_f_*(*x*) denotes the task difficulty degree of the scenario, *P_f_*(*x*) denotes the occurrence probability of the scenario. The long-tail function is as follows in the mobility evaluation.

(9) $$V_{m}(x)=T_{m}(x)\cdot P_{m}(x)$$

where *V_m_*(*x*) denotes the criticality of the scenario, *T_m_*(*x*) denotes the index of the travel efficiency of the scenario, *P_m_*(*x*) denotes the occurrence probability of the scenario. A threshold can be set according to the actual situation. *T_m_*(*x*) will be set to 0 if the travel efficiency exceeds the threshold and will be set to 1 if the travel efficiency is less than the threshold. For the rider’s comfort evaluation, the long-tail function is defined as follows.

(10) $$V_{c}(x)=J_{c}(x)\cdot P_{c}(x)$$

where *V_c_*(*x*) denotes the criticality of the scenario, *J_c_*(*x*) denotes the index of the jerk of the scenario, *P_c_*(*x*) denotes the occurrence probability of the scenario. A threshold can be set according to the actual situation. *J_c_*(*x*) will be set to 1 if the jerk exceeds the threshold and will be set to 0 if the jerk is less than the threshold.

#### 3.2.2. Construction Specifications for the Generalized Scenario Space

The generalized scenario space can be divided into ISSD and NDD. The ISSD can be constructed by the discretization of decision variables, which refer to scenario elements with high-importance weights obtained by the AHP. In the construction of the ISSD ranges of decision variables with relatively high importance weights should be large and their discrete steps should be small to make the ISSD cover as many scenarios as possible. Ranges and discrete steps of decision variables with relatively low importance weights are determined by principles that critical scenarios are included in the ISSD.

The NDD comes from the data of drivers, vehicles, other traffic participants, and the surrounding environment during the real driving process, and is observed and recorded by the advanced data collection system under natural conditions. The NDD reflects the motion characteristics of vehicles and provides a variety of sources of typical traffic scenario data to evaluate the performance of automated vehicles. To ensure the effectiveness of NDD, the following principles should be obeyed in the process of data collection:

- The data collection system should include various kinds of sensors, such as lidar, millimeter wave radar, camera, inertial navigator, and light sensor, to sense the traffic environment comprehensively.
- The data collection area should include different cities and regions, the total mileage should be relatively large.
- The data collection environment should mainly include various road types, weather, and light conditions.

The original data needs to be cleaned, screened, extracted, calibrated, deconstructed, and reconstructed, after that the NDD is obtained.

#### 3.2.3. The Long-Tail Function Construction of the LD-Scenario

The assumption of the LD-Scenario is relatively ideal. It holds that the situation of the scenario can be represented by the states of the ego vehicle and the object vehicle at the critical moment of the scenario. For the LD-Scenario, the unified form of the long-tail function for the safety evaluation can be directly used. To obtain the long-tail function for the safety evaluation, the occurrence probability *P_s_*(*x*) and the risk degree *R_s_*(*x*) need to be solved by the following methods.

The occurrence probability of the scenarios in the ISSD can be solved based on the corresponding NDD. The solution idea is to convert the occurrence probability of the scenario in the NDD into those of four scenarios in the two-dimensional ISSD or eight scenarios in the three-dimensional ISSD which are adjacent to the scenario from the NDD. The CCM is used to realize the transformation and figure out the occurrence probability of scenarios in ISSD.

The CCM is a non-negative linear combination of several variables with assigning different weights to each variable and the sum of all weights is 1 [52]. For example, *X* = {*x_i_*, *i =* 1, 2, *…*, *n*} is a vector of *n* variables, its convex combination *M_X_* is as follows.

(11) $$M_{X}=\sum_{i=1}^{n}\lambda_{i}x_{i}=1(\lambda_{i}\geq 0)$$

where *𝜆_i_* denote the weights of *x_i_*.

CCM is adopted to solve the occurrence probability of scenarios in the discrete step of the ISSD with following assumptions:

- The occurrence probability changes continuously and linearly;
- The continuous and linear law can be reflected by the Euclidean distance between the scenario from NDD and its adjacent scenarios from ISSD;
- The scenario whose Euclidean distance is close to the scenario from NDD will be assigned more weights of occurrence probability, and vice versa.

In the two-dimensional ISSD, the relative distance *DI_D-rd_* and relative speed *DI_D-sp_* between the ego vehicle and object vehicle are chosen as decision variables. For simplicity, *R* is the relative distance and ∆*v* is the relative speed. After determining the range and the discrete step of *R* and ∆*v*, the two-dimensional ISSD can be obtained by the discretization of two decision variables.

**Figure 5:**
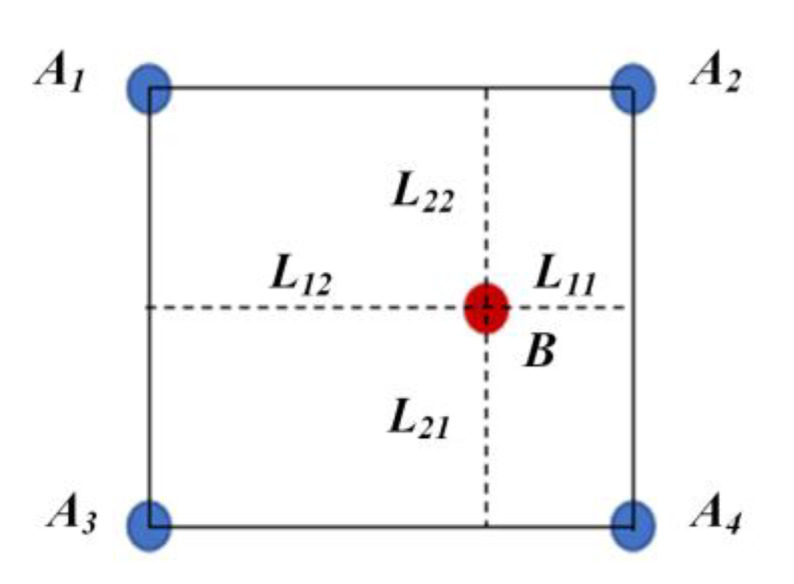
CCM for two-dimensional ISSD.

In Figure 5, a discrete step of two-dimensional ISSD is selected to illustrate CCM. *B*(*R_b_*, ∆*v_b_*) denotes the scenario from the NDD while *A_1_*(*R_a1_*, ∆*v_a1_*), *A_2_*(*R_a2_*, ∆*v_a2_*), *A_3_*(*R_a3_*, ∆*v_a3_*) and *A_4_*(*R_a4_*, ∆*v_a4_*) denote those adjacent to *B* in two-dimensional ISSD. *L_11_*, *L_12_*, *L_21_* and *L_22_* denote the distance from *B* to the edges of the rectangle composed of *A_1_*, *A_2_*, *A_3_* and *A_4_*. As a result, the scenario *B* can be represented by the scenarios *A_1_*, *A_2_*, *A_3_* and *A_4_* through CCM, which can be expressed as follows.

(12) $$B=\sum_{i=1}^{4}\omega_{i}\cdot A_{i}$$

where *ω_i_* are the weights of *A_i_* and they can be expressed as

(13) $$\omega_{1}=\frac{L_{11}}{R_{a2}-R_{a1}}\cdot \frac{L_{21}}{\Delta v_{a2}-\Delta v_{a1}}$$

(14) $$\omega_{2}=\frac{L_{12}}{R_{a2}-R_{a1}}\cdot \frac{L_{21}}{\Delta v_{a2}-\Delta v_{a1}}$$

(15) $$\omega_{3}=\frac{L_{11}}{R_{a2}-R_{a1}}\cdot \frac{L_{22}}{\Delta v_{a2}-\Delta v_{a1}}$$

(16) $$\omega_{4}=\frac{L_{12}}{R_{a2}-R_{a1}}\cdot \frac{L_{22}}{\Delta v_{a2}-\Delta v_{a1}}$$

In the three-dimensional ISSD, the *DI_D-rd_*, *DI_D-sp_* and the relative acceleration *DI_D-ac_* between the ego vehicle and object vehicle are chose as the decision variables. For simplicity, ∆*a* is used to denote the relative acceleration. After determining the range and the discrete step of *R*, ∆*v* and ∆*a*, the three-dimensional ISSD can be obtained by the discretization of the three decision variables.

**Figure 6:**
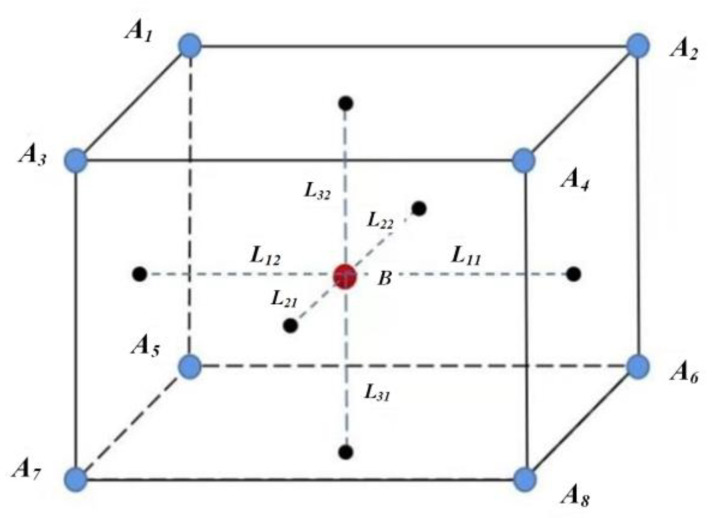
CCM for three-dimensional ISSD.

In Figure 6, the discrete step of the three-dimensional ISSD is selected to illustrate CCM. *B*(*R_b_*, ∆*v_b_*, ∆*a_b_*) denotes a scenario from NDD while *A_1_*(*R_a1_*, ∆*v_a1_*, ∆*a_a1_*), *A_2_*(*R_a2_*, ∆*v_a2_*, ∆*a_a2_*), *A_3_*(*R_a3_*, ∆*v_a3_*, ∆*a_a3_*), *A_4_*(*R_a4_*, ∆*v_a4_*, ∆*a_a4_*), *A_5_*(*R_a5_*, ∆*v_a5_*, ∆*a_a5_*), *A_6_*(*R_a6_*, ∆*v_a6_*, ∆*a_a6_*), *A_7_*(*R_a7_*, ∆*v_a7_*, ∆*a_a7_*) and *A_8_*(*R_a8_*, ∆*v_a8_*, ∆*a_a8_*) denote the scenarios which are adjacent to *B* in the three-dimensional ISSD. *L_11_*, *L_12_*, *L_21_*, *L_22_*, *L_31_* and *L_32_* denote the distance from *B* to the sides of the cube composed of *A_1_*, *A_2_*, *A_3_*, *A_4_*, *A_5_*, *A_6_*, *A_7_* and *A_8_*. As a result, the scenario B can be represented by the scenarios *A_1_*, *A_2_*, *A_3_*, *A_4_*, *A_5_*, *A_6_*, *A_7_* and *A_8_* through CCM.

(17) $$B=\sum_{i=1}^{8}\omega_{i}\cdot A_{i}$$

where *ω_i_* are the weights of *A_i_* and can be expressed as follows.

(18) $$\omega_{1}=\frac{L_{11}}{R_{a2}-R_{a1}}\cdot \frac{L_{21}}{\Delta v_{a2}-\Delta v_{a1}}\cdot \frac{L_{31}}{\Delta a_{a2}-\Delta a_{a1}}$$

(19) $$\omega_{2}=\frac{L_{12}}{R_{a2}-R_{a1}}\cdot \frac{L_{21}}{\Delta v_{a2}-\Delta v_{a1}}\cdot \frac{L_{31}}{\Delta a_{a2}-\Delta a_{a1}}$$

(20) $$\omega_{3}=\frac{L_{11}}{R_{a2}-R_{a1}}\cdot \frac{L_{22}}{\Delta v_{a2}-\Delta v_{a1}}\cdot \frac{L_{31}}{\Delta a_{a2}-\Delta a_{a1}}$$

(21) $$\omega_{4}=\frac{L_{12}}{R_{a2}-R_{a1}}\cdot \frac{L_{22}}{\Delta v_{a2}-\Delta v_{a1}}\cdot \frac{L_{31}}{\Delta a_{a2}-\Delta a_{a1}}$$

(22) $$\omega_{5}=\frac{L_{11}}{R_{a2}-R_{a1}}\cdot \frac{L_{21}}{\Delta v_{a2}-\Delta v_{a1}}\cdot \frac{L_{32}}{\Delta a_{a2}-\Delta a_{a1}}$$

(23) $$\omega_{6}=\frac{L_{12}}{R_{a2}-R_{a1}}\cdot \frac{L_{21}}{\Delta v_{a2}-\Delta v_{a1}}\cdot \frac{L_{32}}{\Delta a_{a2}-\Delta a_{a1}}$$

(24) $$\omega_{7}=\frac{L_{11}}{R_{a2}-R_{a1}}\cdot \frac{L_{22}}{\Delta v_{a2}-\Delta v_{a1}}\cdot \frac{L_{32}}{\Delta a_{a2}-\Delta a_{a1}}$$

(25) $$\omega_{8}=\frac{L_{12}}{R_{a2}-R_{a1}}\cdot \frac{L_{22}}{\Delta v_{a2}-\Delta v_{a1}}\cdot \frac{L_{32}}{\Delta a_{a2}-\Delta a_{a1}}$$

The occurrence probability of scenarios in the ISSD can be obtained based on the CCM, which is proposed to convert all the scenarios in NDD into the scenarios in ISSD. The risk degree of the scenarios in the ISSD can be solved based on the corresponding NDD as well. The two-dimensional ISSD, which is constructed by the discretization of *R* and ∆*v*, usually chooses the time to collision (*TTC*) to represent the risk degree of the scenarios. The smaller the values of *TTC*, the higher the risk degree of the scenarios is. *TTC* is calculated as the ratio of *R* and its change rate as shown in Equation (26).

(26) $$TTC=\frac{R}{\dot{R}}$$

where $\dot{R}$ is the change rate of *R*. The risk degree of the two-dimensional scenarios in the ISSD is shown in Table 4.

**Figure 7:**
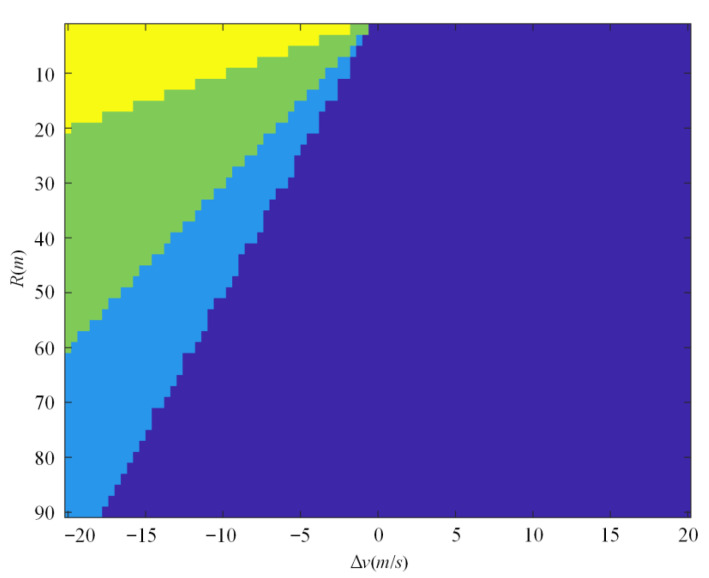
The boundaries of the two-dimensional ISSD according to *TTC*.

Boundaries of two-dimensional ISSD are shown in Figure 7. The yellow, green, pale blue and navy blue regions represent crash, emergency, conflict, and safe scenarios, respectively.

However, when it comes to three-dimensional ISSD, which is usually constructed by the discretization of three decision variables such as the relative distance *R*, the relative speed ∆*v*, and the relative acceleration ∆*a* between the ego vehicle and the object vehicle, it is insufficient to take *TTC* as the only index of the risk degree as the relative acceleration is not considered. To fix it, scenarios from the corresponding NDD can be pre-classified by *TTC*. According to the classification results, the SVR in machine learning (ML) is introduced to obtain the accurate boundaries of the NDD according to the joint distribution of *TTC* and ∆*a*.

SVR is an important application branch of support vector machine (SVM), which is mainly used to solve the problems of regression and prediction [53]. Setting the relative acceleration ∆*a* of the scenarios in the NDD as *x* and *TTC* of the scenarios in NDD as *y*, then the training data set is {(*x_1_*, *y_1_*), (*x_2_*, *y_2_*), …, (*x_n_*, *y_n_*)}. The purpose of SVR is to obtain the regression model *f*(*x*) which is as close as possible to *y* through learning. The regression model is defined as follows.

(27) $$f(x)=\omega^{T}x+b$$

where *ω* denotes the normal vector and determines the direction of the hyperplane, *b* denotes the displacement term which determines the distance between the hyperplane and the origin.

SVR assumes that the acceptable maximum deviation between *f*(*x*) and *y* is *ε*. In other words, the loss will be calculated only when the absolute value of the difference between *f*(*x*) and *y* is greater than *ε*, and the problem can be formalized as follows.

(28) $$\underset{\omega ,b}{\min}\frac{1}{2}{\left\|\omega \right\|}^{2}+c\sum_{i=1}^{m}\ell_{\varepsilon }(f(x_{i})-y_{i})$$

where *c* and $\ell_{\varepsilon }$ are regularization constant and *ε*-insensitive loss function, respectively.

(29) $$\ell_{\varepsilon }(z)=\{\begin{array}{c} 0,\begin{array}{cc} & if\left|z\right|\leq \varepsilon \end{array}\\ \left|z\right|-\varepsilon ,\begin{array}{cc} & otherwise \end{array} \end{array}$$

Equation (29) can be reconstructed as follows.

(30) $$\underset{\omega ,b,\xi_{i},{\hat{\xi }}_{i}}{\min}\frac{1}{2}{\left\|\omega \right\|}^{2}+C\sum_{i=1}^{m}\left(\xi_{i}+{\hat{\xi }}_{i}\right)$$

$$s.t.\;f(x_{i})-y_{i}\leq \varepsilon +\xi_{i},$$

$$y_{i}-f(x_{i})\leq \varepsilon +{\hat{\xi }}_{i},$$

$$\xi_{i}\geq 0,\xi_{i}\geq 0,i=1,2,\ldots ,m.$$

where $\xi_{i}$ and ${\hat{\xi }}_{i}$ are the relax matrices, $\mu_{i}$, ${\hat{\mu }}_{i}$, $\alpha_{i}$ and ${\hat{\alpha }}_{i}$ are the Lagrange multipliers, then the Lagrange function of Equation (31) can be obtained by Lagrange multiplier method.

(31) $$\begin{array}{c} L(\omega ,b,\alpha ,\hat{\alpha },\xi ,\hat{\xi },\mu ,\hat{\mu })\;\;\\ =\frac{1}{2}{\left\|\omega \right\|}^{2}+C\sum_{i=1}^{m}\left(\xi_{i}+{\hat{\xi }}_{i}\right)-\sum_{i=1}^{m}\mu_{i}\xi_{i}-\sum_{i=1}^{m}{\hat{\mu }}_{i}{\hat{\xi }}_{i}\;\;\\ +\sum_{i=1}^{m}\alpha_{i}(f(x_{i})-y_{i}-\varepsilon -\xi_{i})+\sum_{i=1}^{m}{\hat{\alpha }}_{i}(y_{i}-f(x_{i})-\varepsilon -{\hat{\xi }}_{i}) \end{array}$$

Let the partial derivatives of *L* be zero, *ω* can be obtained as follows.

(32) $$\omega =\sum_{i=1}^{m}({\hat{\alpha }}_{i}-\alpha_{i})x_{i}$$

The dual form of SVR can be obtained as follows.

(33) $$\underset{\alpha ,\hat{\alpha }}{\max}\sum_{i=1}^{m}y_{i}({\hat{\alpha }}_{i}-\alpha_{i})-\varepsilon ({\hat{\alpha }}_{i}+\alpha_{i})-\frac{1}{2}\sum_{i=1}^{m}\sum_{j=1}^{m}({\hat{\alpha }}_{i}-\alpha_{i})({\hat{\alpha }}_{j}-\alpha_{j})x_{i}^{T}x_{j}$$

$$\begin{array}{c} s.t.\;\sum_{i=1}^{m}({\hat{\alpha }}_{i}-\alpha_{i})=0,\\ 0\leq \alpha_{i},{\hat{\alpha }}_{i}\leq C. \end{array}$$

Equation (28) can be reconstructed as follows.

(34) $$f(x)=\sum_{i=1}^{m}({\hat{\alpha }}_{i}-\alpha_{i})x_{i}^{T}x+b$$

Solving Equation (34), $\alpha_{i}$ can be obtained. Selecting training data that meet 0 < $\alpha_{i}$ < *c*, *b* can be obtained as follows.

(35) $$b=y_{i}+\varepsilon -\sum_{j=1}^{m}({\hat{\alpha }}_{j}-\alpha_{j})x_{j}^{T}x_{i}$$

If there is no hyperplane solution in the original sample space, the training data can be mapped from the original sample space to a higher dimensional feature space and *ω* and *f*(*x*) need to be reconstructed as follows.

(36) $$\omega =\sum_{i=1}^{m}\left({\hat{\alpha }}_{i}-\alpha_{i})\phi (x_{i}\right)$$

(37) $$f(x)=\sum_{i=1}^{m}({\hat{\alpha }}_{i}-\alpha_{i})\kappa (x,x_{i})+b$$

where *𝜅*(*x, x_i_*) denotes the kernel function and can be expressed as follows.

(38) $$\kappa (x,x_{i})=\phi {\left(x_{i}\right)}^{T}\phi (x_{j})$$

where *𝜙*(*x_i_*) and *𝜙*(*x_j_*) denote the eigenvectors mapped with *x*.

Boundaries of the NDD can be directly mapped into the corresponding ISSD. Finally, the risk degree of the scenarios in the three-dimensional ISSD can be obtained.

#### 3.2.4. The Long-Tail Function Construction of the HD-Scenario

The HD-Scenario is much more complicated than the LD-Scenario as the interaction of the ego vehicle and object vehicle need to be considered. As the total time steps of the scenarios increase, the dimensions of the ISSD will increase, and the scenario number in the ISSD will grow exponentially with the dimension. To reduce the dimension of the ISSD and simplify the computation complexity of the long-tail function for the safety evaluation, the HD-Scenario is regarded as a Markov decision process (MDP) problem.

As the fundamental model in reinforcement learning (RL), MDP is often used to describe and solve large-scale decision-making problems involving uncertainties [54]. States in MDP are those of the relative states between the ego vehicle and object vehicle and actions are the acceleration of the object vehicle. The Markovian property holds that the next action of the object vehicle is only dependent on the current state. The long-tail function of the HD-Scenarios can be constructed as follows.

(39) $$V_{s}(x)=\prod_{i=1}^{n}G(s_{i},a_{i})$$

where *V_s_*(*x*) is the scenario criticality, *n* is the total time step, *s_i_* is the state at time *t*, *a_i_* is the action at time *t*, and *G*(*s_i_, a_i_*) is the value of each state–action pair as follows.

(40) $$G(s_{i},a_{i})=R(s_{i},a_{i})\cdot P(a_{i}|s_{i})$$

where *R*(*s_i_, a_i_*) is the risk degree of the state–action pair, and *P*(*a_i_*|*s_i_*) denotes the occurrence probability of the state–action pair. In order to obtain the long-tail function, *P*(*a_i_*|*s_i_*) and *R*(*s_i_, a_i_*) need to be solved. The occurrence probability of the state–action pairs can be solved based on the CCM with the corresponding NDD. The risk degree of the state–action pairs can be solved by the corresponding NDD as well. In view of the time-varying, high-order nonlinear, and dynamic characteristics of the dynamic scenario, the Hammerstein identification process (HIP) is selected as the risk degree identification model (RDIM). The HIP is composed of static nonlinear links and dynamic linear links in series [55]. The relative states between the ego vehicle and object vehicle and the acceleration of the object vehicle are taken as inputs of the risk degree identification model and the acceleration of the ego vehicle is the output. It can be seen that the RDIM is a multiple-input single-output (MISO) system.

Various functions such as the dead zone function, the S-type function or the saturation function can be selected in the static nonlinear link of the RDIM. The dynamic linear link with Z-transform is expressed as follows.

(41) $$A(z^{-1})O_{p}(k)=B(z^{-1})\cdot z^{-d}\cdot N(k)$$

where *O_p_(k)* denotes the set of the acceleration of the ego vehicle, *N(k)* denotes the output set of the static nonlinear link, *d* is the input delay order, and defined as an integer multiple of the sampling time. *A*(*z*^−1^) and *B*(*z*^−1^) can be obtained as follows.

(42) $$\{\begin{array}{c} A(z^{-1})=1+a_{1}\cdot z^{-1}+\ldots +a_{q}\cdot z^{-q}\\ B(z^{-1})=b_{1}\cdot z^{-1}+\ldots +b_{n}\cdot z^{-n} \end{array}$$

where (*a_1_*,…, *a_q_*) and (*b_1_*,…, *b_n_*) are the coefficients of the dynamic linear link, *q* and *n* are orders of the dynamic linear link.

After being trained by the data from the NDD, the internal parameters of the RDIM represent the intrinsic attributes of the risk degree of the state–action pairs. Therefore, they are taken as the basis of the risk degree assessment. In order to reduce the spatial dimension of the internal parameters as much as possible and improve the calculation efficiency on the premise of expressing the same model characteristics, principal component analysis (PCA) is used to decouple and reduce the dimension of these parameters [56].

Let *H* represent the total dimension of the internal parameters of the RDIM, and *E* represent the number of input data. The set of internal parameters *X* can be expressed as follows.

(43) $$X=\left[\begin{array}{cccc} x_{11} & x_{12} & \cdots & x_{1E}\\ x_{21} & x_{22} & \cdots & x_{2E}\\ \vdots & \vdots & \vdots & \vdots \\ x_{H1} & x_{H1} & \cdots & x_{HE} \end{array}\right]=\left\{x_{i},i=1,2,\ldots ,E\right\}$$

where *x_i_* denotes the internal parameter vectors of the RDIM.

The input of the PCA is *X* and the output is the main component contribution rate. The algorithm is summarized in Algorithm 1.
**Algorithm 1** Algorithm of PCA **Input**: The set of the internal parameters *X* = {*x_1_*,…, *x_E_*}; **Output**: The matrix of the main component contribution rate W* = (*𝜔_1_*,*𝜔_2_*,…, *𝜔_d_*); **Step 1**: Centralizing the set of the internal parameters: *x_i_* = *x_i_* − (*x_1_* + *x_2_* + …+ *x_m_*)/*m*; **Step 2**: Calculating the covariance matrix of the set of the internal parameters: *XX^T^*; **Step 3**: Doing eigenvalue decomposition of the matrix *XX^T^*; **Step 4**: Taking the eigenvector corresponding to the first *d* largest eigenvalues: *𝜔_1_*,…, *𝜔_d_*.

The ratio of the sum of the eigenvalues of the first *d* principal components to the sum of all eigenvalues is defined as the contribution rate of the principal components *M_d_* which can be expressed as

(44) $$M_{d}=\sum_{i=1}^{d}\lambda_{i}/\sum_{i=1}^{E}\lambda_{i}$$

where *𝜆_i_* denotes the eigenvalues.

Considering the general requirement for the range of *M_d_* in the PCA algorithm, the value of *d* corresponding to *M_d_* ≥ 85% is taken as the independent parameter dimension of the model to ensure the dimension reduction effect. The principal component matrix *L* of the RDIM can be expressed as

(45) $$L=\left[\begin{array}{cccc} l_{11} & l_{12} & \cdots & l_{1E}\\ l_{21} & l_{22} & \cdots & l_{2E}\\ \vdots & \vdots & \vdots & \vdots \\ l_{d1} & l_{d1} & \cdots & l_{dE} \end{array}\right]=\left\{l_{i},i=1,2,\ldots ,E\right\}$$

The ant colony clustering algorithm (ACCA) is used to realize the risk degree classification. The ACCA is a heuristic searching algorithm inspired by population optimization [57]. Through the pheromone cooperation mechanism, the searching process is hard to fall into local optimization. The input of the ACCA is the matrix *L*. The risk degree of state–action pairs is divided into four categories as the cluster number: the safe state–action pair, the conflict state–action pair, the emergency state–action pair, and the crash state–action pair. The aim of the algorithm is to find a partitioned way that can minimize the sum of the distance from each sample to the cluster centers, which can be expressed as follows.

(46) $$\min J(\omega ,c)=\sum_{j=1}^{4}\sum_{i=1}^{E_{j}}\sum_{p=1}^{d}\omega_{ij}{\left\|l_{ip}-c_{jp}\right\|}^{2}$$

where *J* denotes the sum of the distance from each sample to the cluster centers, *l_ip_* denotes the *p*th model parameter characteristic value of the *i*th sample, *c_jp_* denotes the *p*th model parameter characteristic value of the *j*th class center as follows.

(47) $$c_{jp}=\sum_{i=1}^{E_{j}}\omega_{ij}l_{ip}/\sum_{i=1}^{E_{j}}\omega_{ij}(j=1,\ldots ,4;p=1,\ldots ,d)$$

where *E_j_* denotes the observation variable corresponding to the *j*-th class, *ω_ij_* denotes the indicator of the affiliation between the observation variable and the classification as follows.

(48) $$\omega_{ij}=\{\begin{array}{c} 1,\;if\;i\;belongs\;to\;the\;category\;j\\ 0,if\;i\;doesn’t\;belong\;to\;the\;category\;j \end{array}(j=1,\ldots ,4;i=1,\ldots ,E_{j})$$

The classification quality of the ACCA can be improved by iterating the update equation, which can be expressed as follow.

(49) $$P_{ij}=\tau_{ij}/\sum_{i=1}^{E_{j}}\tau_{ij}(j=1,\ldots ,4)$$

where *P_ij_* denotes the transition probability, *𝜏_ij_* denotes the standardized pheromone between the sample *i* and the class *j* as follows.

(50) $$\tau_{ij}(t+1)=(1-\rho )\tau_{ij}(t)+\sum_{s=1}^{L}\Delta \tau_{ij}^{s}(i=1,\ldots ,E;j=1,\ldots ,4)$$

where *𝜌* is the pheromone volatility and *t* is the time. Since clustering results do not have real meaning, it is necessary to establish the mapping relationship between the clusters and the risk degree levels. Several representative state–action pairs from each risk degree level are selected as the input of the RDIM and the corresponding internal parameters are clustered, then the mapping relationship between the clusters and the risk degree levels is established.

### 3.3. Critical Scenario Searching Methods

With the scenario boundaries obtained from the long-tail functions, the critical scenario datasets are searched based on the dynamic scenario space search method. In the LD-Scenario generation system, common optimization methods are suitable for extracting critical scenarios, and the key LD-node generation is proposed to search critical scenarios. In order to improve the completeness of critical scenarios in the specific scenario space, the critical sample expansion method is used to enlarge critical scenarios to a specific scale. In the HD-Scenario generation system, the high-dimensional elements have the property that the adjacent quantities of the element sequence are characterized by sequential influence, which is consistent with the Markov process. Therefore, the reinforcement learning-based HD-Scenario generation method is developed to obtain the critical scenarios.

#### 3.3.1. The Critical Scenario Searching Method of the LD-Scenario

As the scenario scale in the ISSD is huge and the criticality values of most scenarios are zero, it is inefficient to search critical scenarios by the traversal searching method. The multi-start optimization method is proposed as the common optimization method and the flood fill method is proposed as the critical sample expansion method. The multi-start optimization algorithm starts a local solver from multiple initial points to sample in the attraction basin where initial points are located and find the local maxima of the long-tail function. It can be used to obtain a number of local critical scenarios with criticality values exceeding the threshold of criticality *𝛾*. *𝛾* can be determined by the target size of critical scenarios. The algorithm is summarized in Algorithm 2.
**Algorithm 2** Algorithm of multi-start optimization **Input**: The long-tail function of the LD-Scenario *V_s_*(*x*); **Output**: Local critical scenarios; **Step 1**: Generating multiple initial points by manually or a space-filling method such as random sampling; **Step 2**: Finding the minimum of the attraction basin where the initial point is located; **Step 3**: Verifying whether these local maxima exceed the threshold of criticality *𝛾*; **Step 4**: Local maxima exceeding *𝛾* are taken as the local critical scenarios.

Flood fill algorithm is applied to find all critical scenarios that exceed *𝛾*. It is a basic one in computer graphics that determines the area connected to a given node in multi-dimensional arrays [58]. The key idea is to explore critical scenarios of unexplored ISSD from local critical scenarios outwards rather than traversal searching all of the ISSD. The algorithm is summarized in Algorithm 3.
**Algorithm 3** Algorithm of flood fill **Input**: The long-tail function of the LD-Scenario *V_s_*(*x*), local critical scenarios, the ISSD; **Output**: All critical scenarios; **Step 1**: Finding scenarios which are adjacent to the critical scenarios in ISSD; **Step 2**: Verifying whether the criticality values of these scenarios exceed *𝛾*; **Step 3**: Those scenarios whose criticality values exceed *𝛾* will be marked as new critical scenarios; **Step 4**: Continue to explore adjacent scenarios of new critical scenarios until all the criticality values of them do not exceed *𝛾*.

#### 3.3.2. The Critical Scenario Searching Method of the HD-Scenario

Critical scenarios can be obtained by the *Q*-learning algorithm, which is a value-based algorithm in RL [59]. The main advantage of the algorithm is that it uses the temporal difference method which integrates the Monte Carlo method and dynamic programming method for offline learning.

*Q*(*s*, *a*) is the expectation of benefits that can be obtained when taking action *a* in the state *s* at a certain time. The environment can feedback on the corresponding reward *r* according to the action of the agent. Therefore, the main idea of the algorithm is to build a *Q*-table that includes the state and the action to store the value of *Q*, and select the action that can obtain the maximum benefits according to the value of *Q*.

The Bellman equation is used to solve the optimal decision strategy of MDP, which can be expressed as follows.

(51) $$V_{\pi }(s)=E_{\pi }[R_{t+1}+\gamma V_{\pi }(S_{t+1})|S_{t}=s]$$

where *𝜋* denotes the strategy, *S* denotes the set of states, *R* denotes the set of rewards, *γ* denotes the discount factor, *t* denotes the time, *s* denotes the state at time *t* and *V_𝜋_*(*s*) denotes the cost function. The updated formula of *Q* can be expressed as follows.

(52) $$Q(S_{t},A_{t})\leftarrow Q(S_{t},A_{t})+\alpha [R_{t+1}+\gamma \underset{a}{\max}Q(S_{t+1},a)-Q(S_{t},A_{t})]$$

where *A* denotes the set of actions, *a* denotes the action at a certain time and *𝛼* denotes the learning rate. By the *Q*-learning algorithm, the best strategy, which is the critical scenario, corresponding to each initial state can be obtained. The algorithm is summarized in Algorithm 4.
**Algorithm4** Algorithm of *Q*-learning **Input**: The environment *E*, the set of actions *A*, the initial state *s_0_*, the discount factor *γ*, the learning rate *α*; **Output**: The optimal strategy π*; **Step 1**: Initializing *Q* and calculating *π*, *Q*(*s*,*a*) = 0, *𝜋*(*s*,*a*) = 1/*A*(*s*); **Step 2**: Initializing *s*, *s* = *s_0_*; **Step 3**: Entering the loop, for *t* = 1, 2,…do **Step 4**:  a′ = *𝜋*(*s′*); **Step 5**:  *Q*(*s*,*a*) = *Q*(*s*,*a*) + α[r + γ*Q*(*s′*,*a′*) − *Q*(*s*,*a*)]; **Step 6**:  *𝜋*(*s*) = arg max *_a_*_″_
*Q*(*s*,*a″*); **Step 7**:  s = *s′* **Step 8**: end for.

## 4. Experiment Verification and Performance Analysis

### 4.1. Field Test Platform for NDD

**Figure 8:**
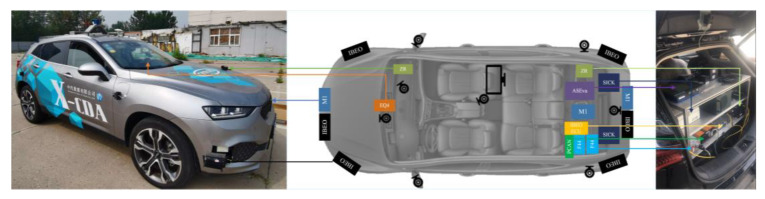
The platform and the layout of the scenario collection system in the platform.

Two vehicles equipped with various types of sensors are used as the data collection platform for NDD. The data collection system mainly includes the on-board controller, the software of scenario collection, the lidar, the lane line sensor, the rainfall and light sensor, the HD camera, the high-precision inertial navigation system and the industrial control microcomputer. The platform and the layout of the data collection system in the platform are shown in Figure 8.

**Figure 9:**
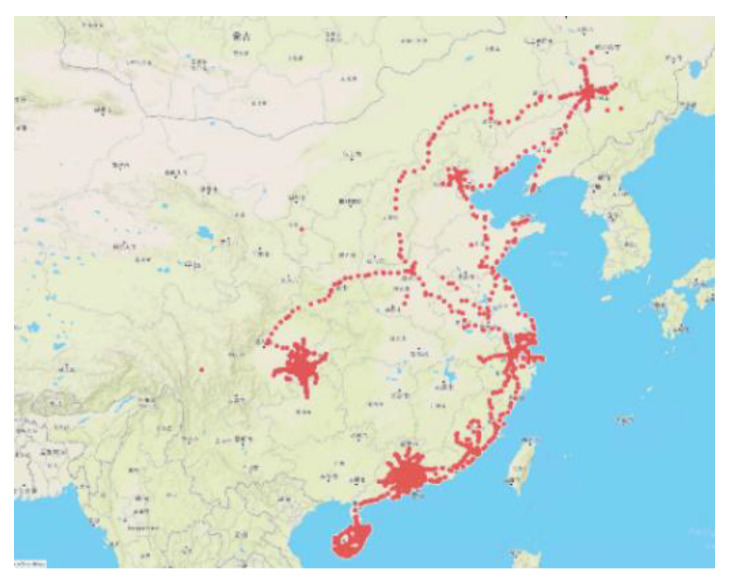
The distribution of the collection areas of the NDD.

The NDD is obtained from the China Automotive Technology and Research Center Co., Ltd. (CATARC). The total mileage of the NDD is 114 thousand kilometers. The collection areas of the NDD cover representative cities in China, including Changchun, Beijing, Tianjin, Shanghai, Guangzhou, Haikou, Chongqing, and the surrounding areas of these cities. These regions have relatively developed economies, large populations, and traffic densities. Therefore, the scenario data collected are rich and diverse enough. The distribution of the collection areas of the NDD is shown in Figure 9.

The device specifications in the scenario collection system are shown in Table 5.

### 4.2. Analysis and Evaluation Results of the LD-Scenario Generation Theory

**Figure 10:**
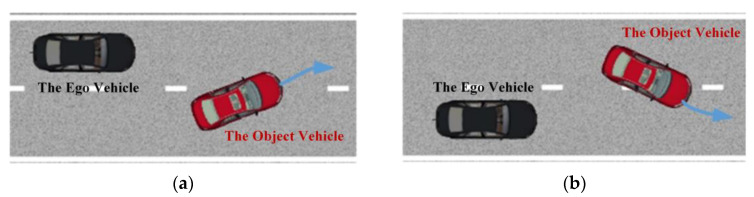
The ODD of the cut-in scenario. The red vehicle denotes the object vehicle and the black one denotes the ego vehicle. (**a**) The object vehicle cuts into the right lane of the ego vehicle; (**b**) the object vehicle cuts into the left lane of the ego vehicle.

To verify the effectiveness of the LD-Scenario generation theory, the cut-in scenario is proposed. The cut-in scenario is one of the most common scenarios in the traffic and prone to cause accidents, so the research on it is of great significance to the testing of automated vehicles. During the cut-in process, the object vehicle drives from the adjacent lane to the lane of the ego vehicle and keep its driving mode in front of the ego vehicle. For each cut-in event, the critical moment is determined by the time instant when the object vehicle crosses the lane marking. It is assumed that the risk degree at the critical moment can represent the risk degree of the cut-in event. The ODD of the cut-in scenario is shown in Figure 10.

#### 4.2.1. The Result of Scenario Element Optimization

When solving the importance weights of scenario elements in the selected ODD, it is not necessary to consider static elements and some dynamic elements as many of them have been determined. The remaining scenario elements are the initial position *DI_I-po_*, the initial speed *DI_I-sp_*, the trigger mode *DI_D-tm_*, the relative distance *DI_D-rd_*, the relative speed *DI_D-rs_* and the relative acceleration *DI_D-ra_*. The effect of these scenario elements on the automatic driving system is analyzed and the passing time of the effect can be obtained by ETM. The passing time of the effect is shown in Table 6. It is recorded as 1 if a scenario element has a direct effect on a certain level of the automatic driving system, otherwise, it is recorded as 0.

The judgment matrix of these scenario elements is:

(53) $$F=\left[\begin{array}{cccccc} 1 & 1 & 3 & 1/4 & 1/3 & 1/3\\ 1 & 1 & 3 & 1/4 & 1/4 & 1/3\\ 1/3 & 1/3 & 1 & 1/6 & 1/6 & 1/5\\ 4 & 4 & 6 & 1 & 1 & 2\\ 3 & 4 & 6 & 1 & 1 & 1\\ 3 & 3 & 5 & 1/2 & 1 & 1 \end{array}\right]$$

Solving the maximum eigenvalue and eigenvector of the judgment matrix, and the importance weights can be obtained by normalizing the eigenvector in Table 7.

The consistency test is conducted, where *C_R_* = 0.0165 < 0.1. It can be seen that importance weights can meet the consistency requirement. Importance weights of *DI_D-rd_*, *DI_D-rs_*, and *DI_D-ra_* are much greater than those of other scenario elements, thus *R*, ∆*v*, and ∆*a* are selected to construct the three-dimensional ISSD. According to the importance weights and actual conditions of the road traffic, the discretization intervals of *R*, ∆*v*, and ∆*a* are set to 2 m, 0.4 m/s*,* and 0.2 m/s^2^. The boundaries of *R*, ∆*v* and ∆*a* are set to (0 m, 90 m], [−20 m/s, 20 m/s] and [−4 m/s^2^, 4 m/s^2^].

#### 4.2.2. The Analysis of the Cut-In Scenarios in NDD

**Figure 11:**
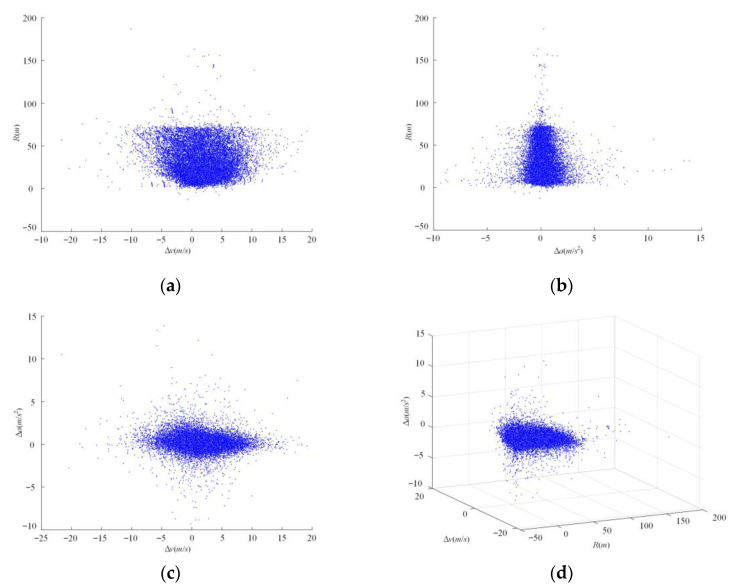
The distributions of 26214 cut-in scenarios obtained from the NDD. (**a**) The joint distribution of *R* and ∆*v* between the ego vehicle and the object vehicle at the critical moment of cut-in scenarios; (**b**) the joint distribution of *R* and ∆*a* between the ego vehicle and the object vehicle at the critical moment of cut-in scenarios; (**c**) the joint distribution of ∆*a* and ∆*v* between the ego vehicle and the object vehicle at the critical moment of cut-in scenarios; (**d**) the joint distribution of *R*, ∆*v* and ∆*a* between the ego vehicle and the object vehicle at the critical moment of cut-in scenarios.

A total of 26,214 cut-in scenarios are obtained from the NDD., *R*, ∆*v* and ∆*a* between the ego vehicle and object vehicle at the critical moment of each cut-in scenario selected from the NDD are extracted in the three-dimensional ISSD. The three-dimensional distribution of cut-in scenarios is shown in Figure 11d. For the convenience of observation, two-dimensional distributions of cut-in scenarios are also constructed in Figure 11a–c. According to these distributions of cut-in scenarios, *R,* ∆*v,* and ∆*a* in cut-in scenarios are mainly concentrated in (0 m, 70 m), (−10 m/s, 10 m/s) and (−3 m/s^2^, 3 m/s^2^), respectively.

#### 4.2.3. The Result of the Occurrence Probability of the Scenarios in ISSD

**Figure 12:**
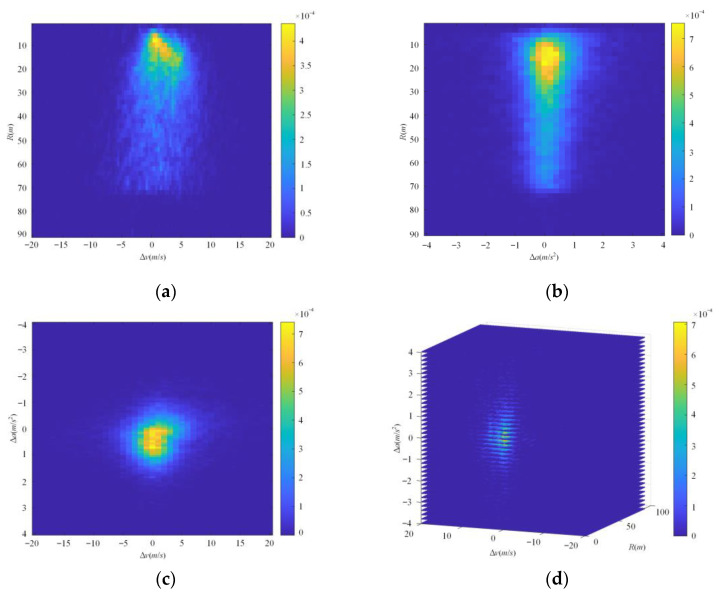
The occurrence probability distributions of the scenarios in ISSD. (**a**) The occurrence probability joint distribution of *R* and ∆*v* between the ego vehicle and the object vehicle at the critical moment of cut-in scenarios; (**b**) the occurrence probability joint distribution of *R* and ∆*a* between the ego vehicle and the object vehicle at the critical moment of cut-in scenarios; (**c**) the occurrence probability joint distribution of ∆*a* and ∆*v* between the ego vehicle and the object vehicle at the critical moment of cut-in scenarios; (**d**) the occurrence probability joint distribution of *R*, ∆*v* and ∆*a* between the ego vehicle and the object vehicle at the critical moment of cut-in scenarios.

The cut-in scenarios in NDD are totally converted into the scenarios in the ISSD and the occurrence probability of scenarios in the three-dimensional ISSD are obtained. The results are shown in Figure 12d. For convenience of observation and comparison, the occurrence probability of the scenarios in the two-dimensional ISSD are also constructed in Figure 12a–c. Results of the three-dimensional ISSD show that *R,* ∆*v*, and ∆*a* of cut-in scenarios with high occurrence probability are mainly concentrated in (10 m, 25 m), (−2.5 m/s, 2.5 m/s), and (−0.5 m/s^2^, 1 m/s^2^), respectively.

#### 4.2.4. The Result of the Scenario Boundaries of NDD and the Risk Degrees of ISSD

**Figure 13:**
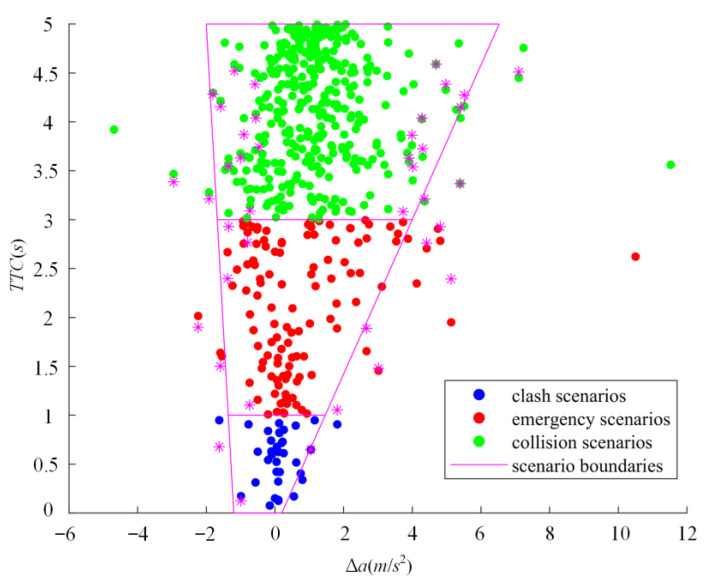
The scenario boundaries of NDD based on the joint distribution of *TTC* and ∆*a*.

According to the scenario classification based on *TTC*, the crash, emergency, and conflict scenarios are selected from the NDD. In the three-dimensional ISSD constructed, it is insufficient to use *TTC* to define the scenario boundary as ∆*a* is not considered. Therefore, the joint distribution consisting of the *TTC* and ∆*a* of crash, emergency, and conflict scenarios is shown in Figure 13.

Critical scenarios are divided into multiple intervals by *TTC*, and extreme points of each interval marked with magenta are solved according to ∆*a*. Extreme points are used as control points to solve scenario boundaries based on SVR. Both maximum and minimum points are taken as the input of SVR, and the linear kernel is used as the kernel function. Scenario boundaries are obtained in Figure 13, and areas enclosed by these boundary boxes represent conflict, emergency, and crash scenario regions, respectively, while the remaining area represents the safe scenario region.

Scenario boundaries can be expressed as follows.

(54) $$\{\begin{array}{c} l_{l}:TTC_{l}=-6.2513\cdot \Delta a_{l}-7.5472\\ l_{r}:TTC_{r}=0.7945\cdot \Delta a_{r}-0.1586\\ l_{m1}:TTC_{m1}=0\\ l_{m2}:TTC_{m2}=1\\ l_{m3}:TTC_{m3}=3\\ l_{m4}:TTC_{m4}=5 \end{array}$$

where *l_l_* and *l_r_* represent the left and right scenario boundaries obtained by SVR, respectively, in Figure 13, *l_m1_*, *l_m2_*, *l_m3_*, and *l_m4_* are obtained by the scenario classification. The scenario classification in the ISSD can be obtained as well. The risk degree corresponding to different levels of the ISSD is set in Table 8.

#### 4.2.5. The Result of the Critical Scenario of LD-Scenario Generation

Values of criticality scenarios in the ISSD can be obtained from the combination of the occurrence probability and risk degree. Setting different thresholds *𝛾* for criticality, and critical scenarios with different sizes can be specified. For example, in the case that *𝛾* = 5.37 × 10^−6^, the multi-start optimization algorithm starts with a random sampling of 100 initial points, and 56 local critical scenarios are obtained. Local critical scenarios are taken as inputs of the flood fill algorithm and critical scenarios in the ISSD with criticality values exceeding *𝛾* are obtained, as shown in Table 9.

**Figure 14:**
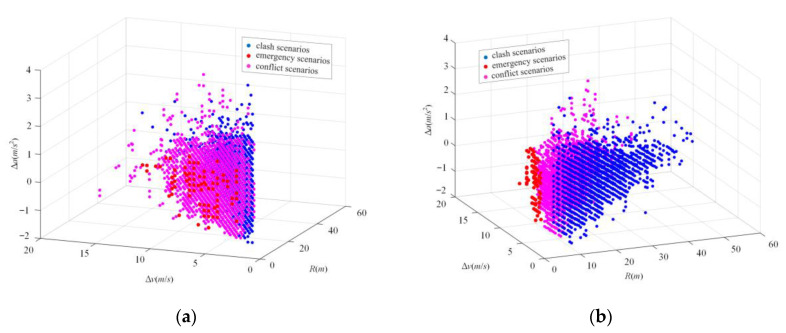
The distribution of critical scenarios in ISSD. (**a**,**b**) show the different perspectives of the distribution.

In the ISSD with 186,345 scenarios, 78 clash scenarios are selected with a proportion of 3.19% in this level and 0.04% in the ISSD; 1363 emergency scenarios are selected with a proportion of 12.6% in this level and 0.73% in the ISSD; 1164 conflict scenarios are selected with the proportion of 7.77% in this level and 0.62% in the ISSD, and no safe scenario is selected. The distribution of critical scenarios in the ISSD is shown in Figure 14. Red points denote clash scenarios, magenta points denote emergency scenarios, and blue points denote conflict scenarios.

#### 4.2.6. The Result of Simulation Tests with Critical Scenarios

**Figure 15:**
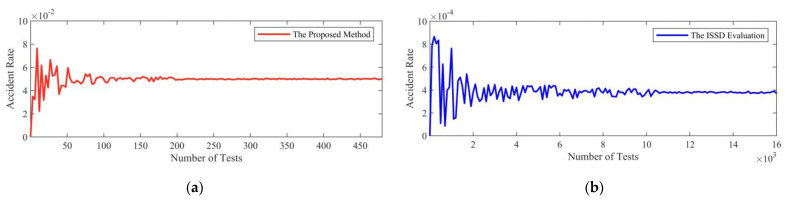
The accident rate of simulation tests of the LD-Scenario. (**a**) The red line denotes the accident rate by the proposed method. (**b**) The blue line denotes the accident rate by the ISSD evaluation method.

To verify the effectiveness of the critical scenarios, a simulated CAV model is evaluated with these scenarios. The simulation model which combines adaptive cruise control (ACC) and autonomous emergency braking (AEB) [6] is adopted. Testing scenarios are sampled from critical scenarios with the *ϵ*-greedy sampling policy, which is commonly used for balancing exploitation and exploration. The policy behaves greedily most of the time, but scenarios in the ISSD which are not critical are selected randomly with the probability *ϵ* as well. The policy is applied with *ϵ* = 0.03. To compare with the proposed method, the CAV model is also tested in scenarios that are sampled uniformly and randomly from the ISSD and called the ISSD evaluation. The accident rate is recorded in Figure 15.

According to simulation test results, the accident rate converges to 5 × 10^−2^ with an 80% confidence level with the proposed method after 100 simulation tests. The accident rate converges to 4 × 10^−4^ with an 80% confidence level after 10^4^ simulation tests with the ISSD evaluation method. The accident rate of the proposed method is 125 times that of the ISSD evaluation method. The accident rate of the proposed method converges 100 times faster than that of the ISSD evaluation method. It shows that the proposed method can significantly improve the accident rate and accelerate the process of the simulation test.

### 4.3. Analysis and Evaluation Results of the HD-Scenario Generation Theory

**Figure 16:**
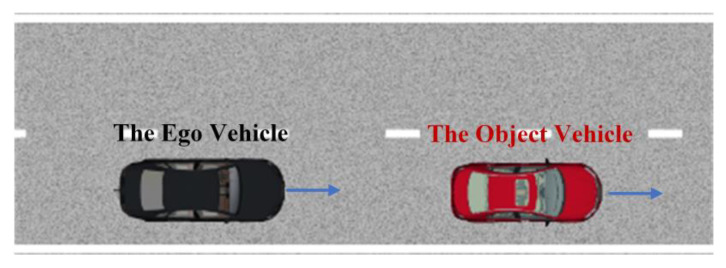
The ODD of the car-following scenario. The red vehicle denotes the object vehicle, and the black vehicle denotes the ego vehicle.

To verify the effectiveness of the HD-Scenario generation theory, the car-following scenario is proposed. The car-following scenario is one of the most common scenarios which refers to the process that the ego vehicle follows the object vehicle for a certain period of time. The ODD of the car-following scenario is shown in Figure 16.

To construct the ISSD, scenario elements with high importance weights, such as the initial speed of the object vehicle, the initial relative distance and speed between the ego vehicle and the object vehicle, and the acceleration profile of the object vehicle are selected as the decision variables *x*. Therefore, it is a typical HD-Scenario. *x* can be expressed as follows.

(55) $$x=[v_{0},R_{0},\Delta v_{0},a_{01,}a_{02},\ldots ,a_{0k}]$$

where *v_0_* denotes the initial speed of the object vehicle, *R_0_* denotes the initial relative distance between the ego vehicle and the object vehicle, ∆*v_0_* denotes the initial relative speed between the ego vehicle and the object vehicle, *a_0_* denotes the acceleration profile of the object vehicle and *k* denotes the total time steps.

The discretization intervals of *v_0_*, *R_0_*, ∆*v_0_* and *a_0_* are set to 2 m/s, 2 m, 2 m/s and 0.2 m/s^2^, respectively. Boundaries of *v_0_*, *R_0_*, ∆*v_0_* and *a_0_* are set to [20 m/s, 40 m/s], (0 m, 90 m], [−20 m/s, 20 m/s] and [−4 m/s^2^, 2 m/s^2^]. *k* is set to 10 which means the car-following scenario lasts for 10 s. Therefore, the scenario number in the ISSD is 21 × 45 × 21 × 31^10^. If these scenarios are directly used to test automated vehicles, it will cause the problem of the “curse of dimensionality”.

To simplify the calculation and reduce the dimension of the ISSD, the car-following scenario is regarded as the MDP. States are relative states of the ego vehicle and the object vehicle, i.e., *v_0_*, *R_0_* and ∆*v_0_*. The action is the acceleration of the object vehicle. It is assumed that the acceleration of the object vehicle is dependent only on its current speed. The number of the state–action pairs is 21 × 45 × 21 × 31 = 615,195, which is much smaller than the number of the scenarios in the ISSD.

To obtain the risk degree of the state–action pairs, the speed of the object vehicle, *R*, ∆*v* and ∆*a* are selected from the NDD of the car-following scenario and used as inputs of the risk-degree identification model. The acceleration of the ego vehicle is used as the output of the model. S-type function is adopted for the static nonlinear link. In the dynamic linear link, both *q* and *n* are set to 3. The input delay order *d* is set to 1. Results show that the average identification accuracy of the RDIMl is 94.47% and the average prediction accuracy is 93.61%. After clustering by ACCA, the state–action pairs with different levels of the risk degree are obtained in Table 10.

**Figure 17:**
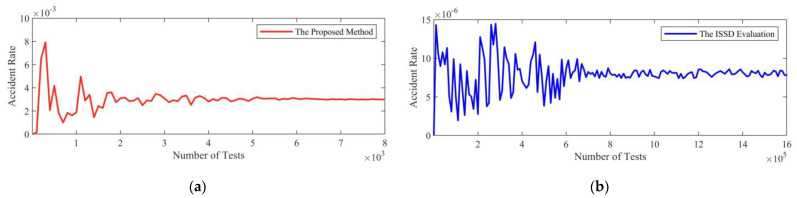
The accident rate of simulation tests of the HD-Scenario. (**a**) The red line denotes the accident rate by the proposed method. (**b**) The blue line denotes the accident rate by the ISSD evaluation method.

A critical car-following scenario should start from a dangerous state, so states from conflict and emergency state–action pairs can be initial states in *Q*-learning, respectively. The best strategy in the *Q*-learning (i.e., the critical scenario) corresponding to each initial state can be obtained. Similar to the method of the simulation in the LD-Scenarios, critical scenarios are sampled with the *ϵ*-greedy sampling policy, and the ISSD evaluation method is also used as a comparation. The *ϵ*-greedy sampling policy is applied with *ϵ* = 0.08 and the accident rate is shown in Figure 17.

From the results of simulation tests, the accident rate converges to 3 × 10^−3^ with an 80% confidence level by the proposed method after 4 × 10^3^ simulation tests. By the ISSD evaluation method, the accident rate converges to 8 × 10^−6^ with an 80% confidence level after 7 × 10^5^ simulation tests. The accident rate of the proposed method is 375 times that of the ISSD evaluation method. The accident rate of the proposed method converges 175 times faster than that of the ISSD evaluation method. It shows that the proposed method can significantly improve the accident rate and accelerate the process of the simulation test.

## 5. Conclusions

The PIHT-based dynamic scenario library generation for automated vehicles is proposed to improve the system performance of TES, in particular, the testing efficiency and accuracy for automated vehicles. A general generation framework of DSL is established based on the physics–intelligence hybrid theory to improve the testing efficiency and accuracy for automated vehicles. The dimension optimization method-based parameterized scenario specification is established. The scenario is parameterized and extracted as the set of multiple heterogeneous elements and the analytic hierarchy process is used in the dimension optimization. Long-tail function construction methods are proposed to obtain clear and reasonable scenario boundaries for both low- and high-dimensional scenarios. The NDD-based ISSD is classified and clustered, and the labeled scenario space boundary is mapped with the critical degree. Critical scenario searching methods are proposed to generate critical scenarios in the dynamic scenario space. The combination method of node optimization and sample expansion is proposed for the generation of low-dimension scenarios and the reinforcement learning method for that of the high-dimension scenarios. Results show that the accident rate of the proposed method is 125 times that of the ISSD evaluation method and converges 100 times faster than that of the ISSD method in the LD-Scenario. In the HD-Scenario, the accident rate of the proposed method is 375 times that of the ISSD evaluation method and converges 175 times faster than that of the ISSD method. It means that the proposed method can significantly improve the accident rate and accelerate the process of the simulation test.

Although this work has achieved some positive results, there are still some areas that can be further improved. First, this paper only takes the cut-in scenario and the car-following scenario as examples to introduce the dynamic scenario library generation of the LD-Scenario and the HD-Scenario. Second, this paper mainly focuses on safety evaluation, for higher-level requirements, functionality, and mobility and the rider’s comfort should be considered in the evaluation scope. In future studies, more types of scenarios and evaluation metrics will be investigated.

## Figures and Tables

**Table 1:** The content of the scaling method.

Relative Ratio	Meaning
1	The two indices are of the same importance
3	One index is slightly important than the other
5	One index is evidently important than the other
7	One index is intensively important than the other
9	One index is absolutely important than the other
2, 4, 6, 8	The median situations of the above

**Table 2:** The corresponding relationship between the transmission number and the relative ratio.

**The Difference of *T*(*q*)**	[0, 2.5)	[2.5, 5)	[5, 7.5)	[7.5, 10)	[10, 12.5)
**The Relative Ratio**	1	2	3	4	5
**The Difference of *T*(*q*)**	[12.5, 15)	[15, 17.5)	[17.5, 20)	[20, 23)	[23, ∞)
**The Relative Ratio**	6	7	8	9	9

**Table 3:** The value of *R_I_*.

***r***	1	2	3	4	5	6	7	8	9
***R_I_***	0	0	0.58	0.90	1.12	1.24	1.32	1.41	1.45

**Table 4:** The risk degree of the scenarios.

*TTC*	The Risk Degree of Scenarios
0 ≤ *TTC* < 1	Crash scenarios
1 ≤ *TTC* < 3	Emergency scenarios
3 ≤ *TTC* ≤ 5	Conflict scenarios
*TTC* < 0 or *TTC* > 5	Safe scenarios

**Table 5:** The device specifications in the scenario collection system.

The Devices in the Scenario Collection System	The Models of the Devices
The controller	ZR-ASFUS-H300
The software of scenario collection	ASEva 3.0
The lidar and its fusion components	IBEO Fusion system, SICK
The lane line sensor	Mobileye EQ4
The rainfall and light sensor	ZR-ILLU-01
The HD camera	AXIS F44 MAIN UNIT
The high-precision inertial navigation system	Novatel CPT-7
The industrial control microcomputer	M1

**Table 6:** The passing times of the effect.

Scenario Elements	Hardware Perception	Task Decision	Path Planning	Control Execution	Passing Times
*DI_I-po_*	1	0	1	1	13
*DI_I-sp_*	0	1	1	0	12
*DI_D-tm_*	0	0	1	1	7
*DI_D-rd_*	1	1	1	1	21
*DI_D-rs_*	0	1	1	1	20
*DI_D-ra_*	0	1	1	1	18

**Table 7:** The importance weights of the scenario elements.

**Scenario Elements**	*DI_I-po_*	*DI_I-sp_*	*DI_D-tm_*	*DI_D-rd_*	*DI_D-rs_*	*DI_D-ra_*
**Important Weights**	0.0853	0.0817	0.0383	0.3131	0.2634	0.2181

**Table 8:** The risk degree and the number of scenarios corresponding to different levels of ISSD.

The Levels of ISSD	The Risk Degree	The Number of Scenarios	The Proportion in ISSD
Clash scenarios	1	2446	1.31%
Emergency scenarios	0.7	10,783	5.79%
Conflict scenarios	0.3	14,980	8.04%
Safe scenarios	0	158,136	84.86%

**Table 9:** The critical scenarios in ISSD.

The Levels of ISSD	The Number of Scenarios	The Proportion in the Level	The Proportion in ISSD
Clash scenarios	78	3.19%	0.04%
Emergency scenarios	1363	12.6%	0.73%
Conflict scenarios	1164	7.77%	0.62%
Safe scenarios	0	0	0

**Table 10:** The risk degree and the number of state–action pairs corresponding to different levels.

The Levels of State–Action Pairs	The Risk Degree	The Number of State–Action Pairs
Clash state–action pairs	1	6235
Emergency state–action pairs	0.7	37,846
Conflict state–action pairs	0.3	87,369
Safe state–action pairs	0	483,745

## Data Availability

Not applicable.
